# Prevalence and potential influencing factors of social frailty among community-dwelling older adults in China: systematic review and meta-analysis

**DOI:** 10.1186/s12877-026-07600-w

**Published:** 2026-06-27

**Authors:** Jinwei Tang, Chen Shen, Ling Wu, Chun Gao, Huiping Xue

**Affiliations:** 1 Department of Nursing, Affiliated Hospital of Nantong University, Nantong, 226001 China; 2 School of Nursing and Rehabilitation, Nantong University, Nantong, 226001 China

**Keywords:** Older adults, Community-dwelling, Social frailty, Risk Factor, Systematic Review, Meta-Analysis

## Abstract

**Objective:**

Based on the local context in China, this study systematically reviews and explores the prevalence of social frailty among community-dwelling older adults in China and its potential influencing factors.

**Methods:**

A systematic search was conducted in databases including CNKI, VIP, Wanfang Data, the Chinese Biomedical Literature Database, PubMed, the Cochrane Library, Web of Science, and Embase, covering the period from the inception of each database to November 9, 2025. Two researchers independently performed the systematic literature search, data extraction, and article quality assessment. Meta-analysis was conducted using Stata 15.1 software, incorporating cross-sectional and cohort studies; a random-effects model was used to calculate the pooled prevalence of social frailty and pre-frailty; odds ratios (OR) and 95% confidence intervals (CI) were used to assess associated influencing factors; the I^2^statistic was used to assess heterogeneity, and subgroup analysis and meta-regression were performed.

**Results:**

An initial search identified 5,488 articles; after screening, a total of 24 studies (*n* = 242,591) were ultimately included. The random-effects model showed that the pooled prevalence of social frailty among community-dwelling older adults in China was 25.0% (95% CI: 19.4%–31.0%, I^2^ = 99.47%, *P* < 0.001),and the prevalence of pre-social frailty was 42.0% (95% CI: 37.6%–46.3%, I^2^ = 94.80%, *P* < 0.001). Gender (female), advanced age (≥ 85 years), depression, physical frailty, housing satisfaction, marital status, and physical frailty were significantly associated with the risk of social frailty (OR: 1.36–4.15).

**Conclusions:**

Pre-social frailty and social frailty are common health challenges faced by community-dwelling older adults in China. Recent data indicate that the prevalence of these conditions remains high; although the prevalence is the very high heterogeneity and should be interpreted with caution, it nevertheless underscores the necessity and urgency of implementing effective interventions. Early identification and intervention for individuals at risk of social frailty are of critical importance for advancing the Healthy China strategy and achieving the goals of active aging.

**PROSPERO registration number:**

CRD420251246136.

**Supplementary Information:**

The online version contains supplementary material available at 10.1186/s12877-026-07600-w.

## Introduction

The global population is rapidly aging, and China’s population aged 60 and older continues to grow. According to the Seventh National Population Census in 2020, China’s population aged 60 and older reached 264 million, accounting for 18.7% of the total population, with the aging process continuing to deepen [1]. Against the backdrop of intensifying global population aging, frailty has emerged as a key public health issue. Frailty is a widely used concept describing a complex state of increased vulnerability resulting from adverse health outcomes associated with aging. In recent years, the concept of frailty has evolved from a narrow focus on physiological frailty to a more comprehensive framework encompassing multiple dimensions—including physiological, psychological, and social aspects [2]. In 2017, Bunt [3] and colleagues first proposed the concept of social frailty, defined as a continuous state of risk in which an individual is at risk of losing, or has already lost, the essential resources, activities, or capabilities required to meet their basic social needs. Social frailty is a dynamic and multidimensional concept; although its manifestations vary among individuals across different cultural and social contexts, its core mechanisms remain consistent. In recent years, research has begun to focus on social frailty among older adults in Chinese communities; however, due to differences in sample sources, measurement tools,and geographical distribution, existing studies have reported inconsistent findings regarding prevalence and influencing factors, and there is a lack of systematic integration. Given China’s unique family structures characterized by a low birth rate and “hollowed-out”households, as well as its urban–rural dichotomy and rapid urbanization, the prevalence of social frailty and its associated factors may exhibit distinct local characteristics. Therefore, we believe there is a need to conduct a systematic review and meta-analysis among community-dwelling older adults in China to comprehensively synthesize existing evidence, clarify the current status of social frailty and its primary influencing factors, and provide evidence-based support for the development of targeted intervention strategies. Consequently, this study aims to address three questions: ① What is the combined prevalence of social frailty and pre-frailty among community-dwelling older adults in China?② Do prevalence rates vary by gender, region, urban/rural status, and time period? ③ Which factors are significantly associated with social frailty?

## Methods

The study protocol has been registered on PROSPERO (CRD420251246136) and was conducted in accordance with the PRISMA [4] and MOOSE [5] guidelines.

### Search strategies

We systematically searched databases including CNKI, VIP, Wanfang Data, CBM, PubMed, Cochrane Library, Web of Science, and Embase from their inception to November 9, 2025. Details of the search strategy were as follows: (Social frailty OR Social vulnerability OR social exclusion OR social isolation) AND (Frailty OR Frailty syndrome) AND (Aged OR Elderly OR Older adult OR Older adults).

### Selection criteria

Inclusion and exclusion criteria: (a) Study design includes cross-sectional or cohort studies; (b) Participants are community-dwelling older adults in China (excluding hospitalized or nursing home residents), aged ≥ 60 years;(c) Outcome measures focused on the prevalence of social frailty and its potential determinants; (d) Diagnostic criteria: Social frailty must be diagnosed using validated instruments such as the Makizako Social Frailty Index (MSFI), Help, Activity, Loneliness, Economy, and Conversation Scale (HALEFT),the Tilbury Frailty Index (TFI), Social Frailty Index (SFI), Social Frailty Screening Questionnaire (SFSQ), Social Frailty Questionnaire (SFQ), Social Vulnerability Index (SVI), Lubben Social Network Scale (LSNS-6), and Bunt; (e) Relevant studies were included regardless of literature quality. Exclusion criteria: (a) Literature types such as reviews, conference papers, or abstracts; (b) Duplicate publications; (c) Literature where full text or required data were unavailable;

### Study selection

Two researchers independently screened the literature. When disagreements arose, a third researcher was consulted. The process was as follows: first, duplicate documents were removed; then, titles and abstracts were reviewed to exclude obviously irrelevant studies; next, full texts of remaining studies were examined; finally, studies meeting inclusion criteria were selected. All disagreements were resolved through tripartite discussion. Data were extracted using a standardized form, including: study authors, publication year, Chinese region, mean age, study type, sample size, sample source, prevalence of social frailty/pre-frailty, influencing factors, and assessment tools.

### Quality assessment

Two researchers assessed the quality of included cross-sectional and cohort studies using the Agency for Healthcare Research and Quality (AHRQ) recommended cross-sectional study evaluation tool and the Newcastle–Ottawa Scale (NOS), respectively. Disagreements were resolved through discussion involving a third researcher. The AHRQ tool comprises 11 items answered as "yes," "no," or "unclear," with "yes" scoring 1 point and "no" or "unclear" scoring 0 points, yielding a total range of 0–11 points. The NOS tool evaluates cohort studies across 8 items, with a maximum score of 9 points, categorizing studies as poor, fair, or good quality. Based on the quality scores, the included studies were classified into three categories: low risk of bias (AHRQ ≥ 8 or NOS ≥ 7), moderate risk of bias (AHRQ 4–7 or NOS 4–6), and high risk of bias (AHRQ ≤ 3 or NOS ≤ 3).

### Statistical analysis

In addition, this study analyzed the prevalence of social frailty among older adults in the community and its 95% confidence interval (CI). We calculated pooled odds ratios (OR) and 95% CIs, identified factors associated with social frailty, and tested for heterogeneity using Stata 15.1. If the I^2^ statistic was less than 50%, the heterogeneity was classified as moderate or low, and a fixed-effects model was used for analysis; if I^2^ was 50% or higher, a random-effects model was used. The significance level for the meta-analysis was set at α = 0.05. To investigate heterogeneity, we predefined the following subgroup analyses: gender, study design, social frailty diagnostic tool, study quality, sample size, region of sample origin, Chinese region, and publication year. Differences between subgroups were compared using the Q-test. Furthermore, a random-effects meta-regression model was employed to examine the effects of publication year, study design, type of diagnostic tool, and sample size on heterogeneity.

## Results

### Study selection

**Fig. 1:**
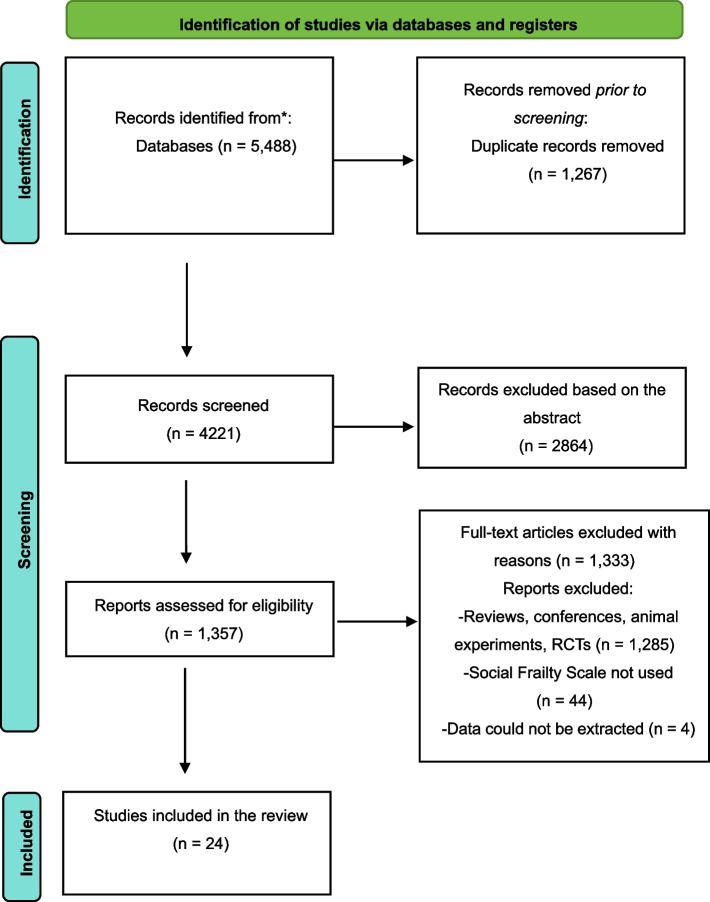
Study selection flowchart

The initial search yielded 5,488 relevant articles from sources including CNKI (*n* = 262), VIP (*n* = 333), Wanfang (*n* = 27), China Biomedical Literature Database (*n* = 38), PubMed (*n* = 594),Web of Science (*n* = 4064), Embase (*n* = 130), and the Cochrane Library (*n* = 40). After removing 1267 duplicates, 2864 studies were excluded based on title and abstract screening. Following full-text review, an additional 1333 studies were excluded, resulting in 24 studies ultimately included in the meta-analysis. The specific screening process is illustrated in Fig. 1.

### Study characteristics

A total of 24 studies were included, and their basic characteristics are summarized in Table 1. These studies were published between 2018 and 2025, covering communities or rural areas across China, and involved a total of 242,591 participants. The study designs included 20 cross-sectional studies and 4 cohort studies, with sample sizes ranging from a minimum of 188 to a maximum of 222,179 participants. Various tools were used to diagnose social frailty across the studies, including the HALFT, MSFI, SFSQ, SFI, TFI, and SVI. The HALFT, MSFI, SFI, and SFSQ are all diagnostic tools developed based on the theory of social need satisfaction; among these, the HALFT scale was the most widely used, with 15 studies employing this tool.

**Table 1:** Characteristics of included studies

Authors	Year	Region in China	Mean Age	Study Type	Sample Size	Sample Source	Number of men and women	Prevalence of social frailty	Prevalence of Pre-Social Frailty	Social Frailty Diagnostic Tool	influencing factors	Quality assessment score
Qi X [6]	2023	Nationwide	≥ 60	Cross-sectional study	222,179	Rural, Community	Male: 116,038 Female: 106,141	15.2%		HALFT	a, b, c, f	7
Sun, Y [7]	2025	East China	≥ 60	Cross-sectional study	1,453	Rural, Community	Male: 662 Female: 791	14.5%	31.5%	HALFT		7
Hu X [8]	2024	East China	71.73 ± 6.4	Cross-sectional study	1175	Mixed	Male: 530 Female: 645	15.7%		HALFT		8
Qin X [9]	2025	East China	70.82 ± 7.432	Cross-sectional study	1611	Mixed	Male: 930 Female: 681	44.2%		HALFT		7
ZeKun Chen [10]	2021	East China	75.3 ± 3.9	Cohort study	1764	Rural	Male: 944 Females: 820	3.5%	36.8%	HALFT		7
Li Zhixiao [11]	2024	Central China	≥ 60	Cross-sectional study	295	Community	Male: 136 Female: 159	29.8%	53.9%	HALFT		9
L. MA [12]	2018	North China	≥ 60	Cohort study	1,697	Community	Male: 193 Female: 249	7.7%	45.6%	HALFT		7
Sun [13]	2023	Southwest	≥ 60	Cooperative study	460	Community	Mixed	24.3%		MSFI	b	9
Wu [14]	2023	East China	76.5 ± 6.9	Cross-sectional study	1,251	Rural	Male: 280 Females: 971	21.7%	34.7%	Bunt		5
Chen [15]	2023	East China	70.33 ± 5.357	Cross-sectional study	208	Community	Male: 83 Females: 125	45.7%		SFSQ		6
Yu Shan [16]	2025	Other regions	70.80 ± 7.48	Cross-sectional study	1826	Rural and community	Male: 837 Females: 989	62.3%		SFI	a, d	5
Hao Juan [17]	2022	Central China	≥ 60	Cross-sectional study	454	Rural, Community	Men: 192 Women: 262	11.2%	54.4%	HALFT	a, b, c, d	5
Zhang Wenya [18]	2021	Central China	Socially Frail Group: 71.4 ± 6.4 Non-socially frail group: 68.3 ± 6.0	Cross-sectional study	359	Rural, community	Male: 149 Females: 210	10.9%		HALFT		5
Qin Yanmei [19]	2025	North China	≥ 60	Cross-sectional study	1,450	Community	Male: 702 Female: 748	38.6%		HALFT	e	5
Zhao Linlin [20]	2025	North China	74.09 ± 6.03	Cross-sectional study	919	Community	Male: 329 Female: 590	11.2%		HALFT	e, f	4
Liu Yue [21]	2024	East China	73.61 ± 5.73	Cohort study	300	Rural	Male: 139 Female: 161	22.3%	41.0%	SFS-8		8
Tang Long [22]	2025	Other regions	≥ 60	Cross-sectional study	235	Community	Men: 64 Female: 171	57.9%		SFI		8
Liu Linfeng [23]	2024	Southwest	≥ 60	Cross-sectional study	215	Community	Male: 121 Female: 94	16.3%		HALFT		4
Chen Yingwei [24]	2022	Southwest	73.8 ± 5.6	Cross-sectional study	2,987	Rural, community	Male: 1,358 Females: 1,629	25.9%	41.4%	HALFT		6
Peng Xinyu [25]	2021	Central China	≥ 60	Cross-sectional study	313	Community	Male: 184 Female: 129	15.7%	41.2%	HALFT		6
Liu Hang [26]	2022	Central China	≥ 60	Cross-sectional study	194	Community	Male: 104 Female: 90	39.2%		TFI		5
Song Ge [27]	2022	North China	Socially Frail Group 74.55 ± 6.34 Non-Socially Frail Group 73.22 ± 5.33	Cross-sectional study	516	Community	Male: 224 Female: 292	12.8%		HALFT		7
Bai Huiqiong [28]	2023	North China	71.57 ± 6.45	Cross-sectional study	188	Community	Male: 83 Females: 105	65.4%		SVI-C		6
Zhang Yuxi [29]	2025	East China	71.69 ± 6.54	Cross-sectional study	542	Community	Male: 267 Female: 275	30.1%		SFI		5

a: Gender; b: Marital status; c: Satisfaction with living environment; d: Depression; e: Physical frailty; f: Age (≥ 85 years vs 65–69 years)

### Methodological quality

Table 1 presents the results of the methodological quality assessment for all 24 studies. Among the cross-sectional studies, 3 were rated as having a low risk of bias, and 17 were rated as having a moderate risk of bias. Among the cohort studies, all 4 were rated as having a low risk of bias. The most common causes of risk of bias included failure to report non-response rates and failure to specify the number and causes of missing data.

### Prevalence of social frailty and social pre-frailty

A total of 24 studies involving 242,591 participants were included in the meta-analysis of the prevalence of social frailty. The prevalence of social frailty reported in the studies ranged from 3.5% to 65.4%. Based on a random-effects model, the pooled prevalence of social frailty among community-dwelling older adults in China was 25% (95% CI: 19.4%–31.0%). There was significant heterogeneity among the included studies (I^2^ = 99.47%, Q = 4,891.648, *P* < 0.001). Nine studies reported the prevalence of pre-social frailty, with estimates ranging from 31.5% to 54.4%. The random-effects model indicated a pooled prevalence of pre-social frailty of 42% (95% CI: 37.6%–46.3%), with significant heterogeneity also observed among studies (I^2^ = 94.80%, Q = 64.705, *P* < 0.001).

### Subgroup analysis

To identify sources of heterogeneity, subgroup analyses were conducted based on sample size, gender, study design, region of sample origin, Chinese region, publication year, study quality, and diagnostic criteria for social frailty; the results are shown in Table 2. Additionally, meta-regression analyses were performed (Table 3). Univariate and multivariate meta-regression analyses indicated that publication year (β = 0.167, 95% CI = 0.034–0.300, *P* = 0.016) and diagnostic tool (β = 0.273, 95% CI = 0.018–0.527, *P* = 0.037) were significantly associated with the prevalence of social frailty; the remaining factors did not show significant associations.

**Table 2:** Subgroup analysis

Subgroup	Number of studies	Prevalence of social frailty	95% CI	I^2^	*P*-value
Gender					
Male	10	22.6	15.1–30.1	99.4	< 0.001
Female	10	22.7	14.9–30.5	99.5	< 0.001
Study Design					
Cross-sectional study	20	29.0	23.1–34.9	99.4	< 0.001
Cohorte study	4	14.0	7.4–20.6	98.2	< 0.001
Social Frailty Diagnostic Tool					
Specific Social Frailty Scale	22	24.2	19.6–28.9	99.5	< 0.001
Other scales	2	52.4	47.6–57.3	< 0.001	< 0.001
Quality of the literature					
Low risk of bias	7	22.5	14.9–30.2	98.9	< 0.001
Moderate risk of bias	17	28.1	21.5–34.7	99.4	< 0.001
Sample size					
> 500	13	23.3	17.2–29.4	99.7	< 0.001
≤ 500	11	30.6	21.1–40.0	97.9	< 0.001
Sample source region					
Community	20	25.6	21.1–30.1	99.0	< 0.001
Rural	10	29.9	20.1–39.7	99.78	< 0.001
China					
Central China	6	19.5	14.5–24.5	94.5	< 0.001
East China	9	23.1	17.1–29.0	99.4	< 0.001
North China	6	24.4	16.7–32.0	99.3	< 0.001

**Table 3:** Results of multiple regression analysis

Covariates	β	SE	95% CI	P
Publication Date	0.167	0.064	0.034–0.300	0.016
Study Design	−0.087	0.080	−0.256—0.081	0.290
Diagnostic Tools	0.273	0.122	0.018—0.527	0.037
Sample size	−0.035	0.063	−0.167—0.096	0.582

### Influencing factors associated with social frailty

A total of 3 studies analyzed gender, 2 addressed depression, 2 focused on housing satisfaction, 2 examined age groups (≥ 85 years vs. 65–69 years), 3 analyzed marital status, and 2 studied physical frailty. The meta-analysis results revealed that gender, depression, housing satisfaction, age, marital status, and physical frailty are the primary influencing factors for social frailty among community-dwelling older adults; specific data are presented in Table 4.

**Table 4:** Results of the meta-analysis on influencing factors for social frailty among community-dwelling older adults

Correlates	Number of Studies	OR (95% CI)	*P*-value	Analytical Model	Heterogeneity	
					p-value	I^2^
Gender	3	1.36 [1.30–1.43]	< 0.001	Fixed	0.350	4.9%
Depression	2	2.82 [1.93–4.10]	< 0.001	fixed	0.796	0%
Housing Satisfaction	2	2.13 [2.01–2.24]	< 0.001	fixed	0.990	0%
Age (≥ 85 years vs 65–69 years)	2	3.25 [1.69–6.26]	< 0.001	fixed	0.844	0%
Marital Status	3	4.32 [4.11–4.54]	< 0.001	random	0.0002	80.0%
Physical Weakness	2	4.15 [1.20–14.39]	0.025	random	0.001	90.7%

### Sensitivity analysis and publication bias

**Fig. 2:**
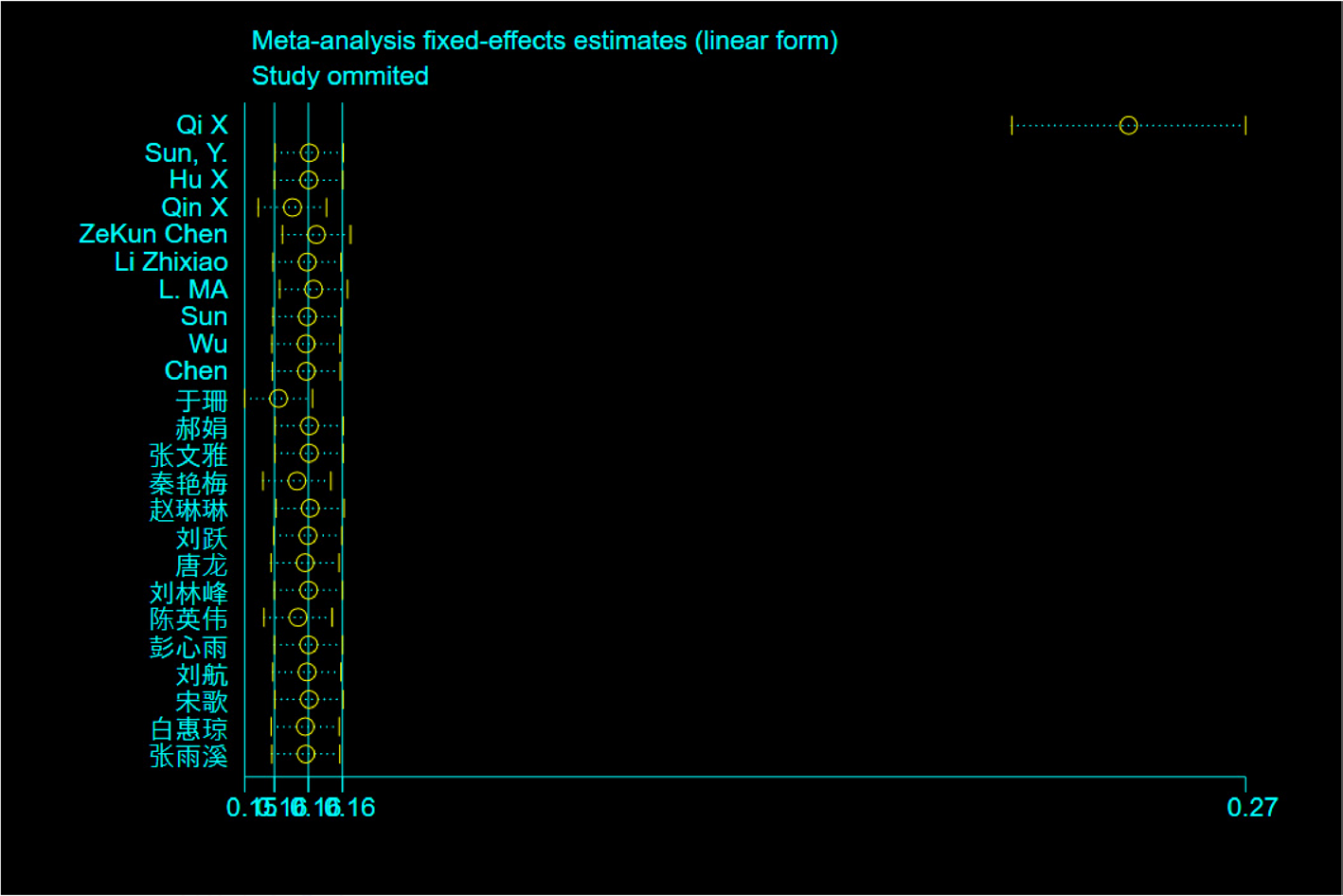
Sensitivity Analysis

**Fig. 3:**
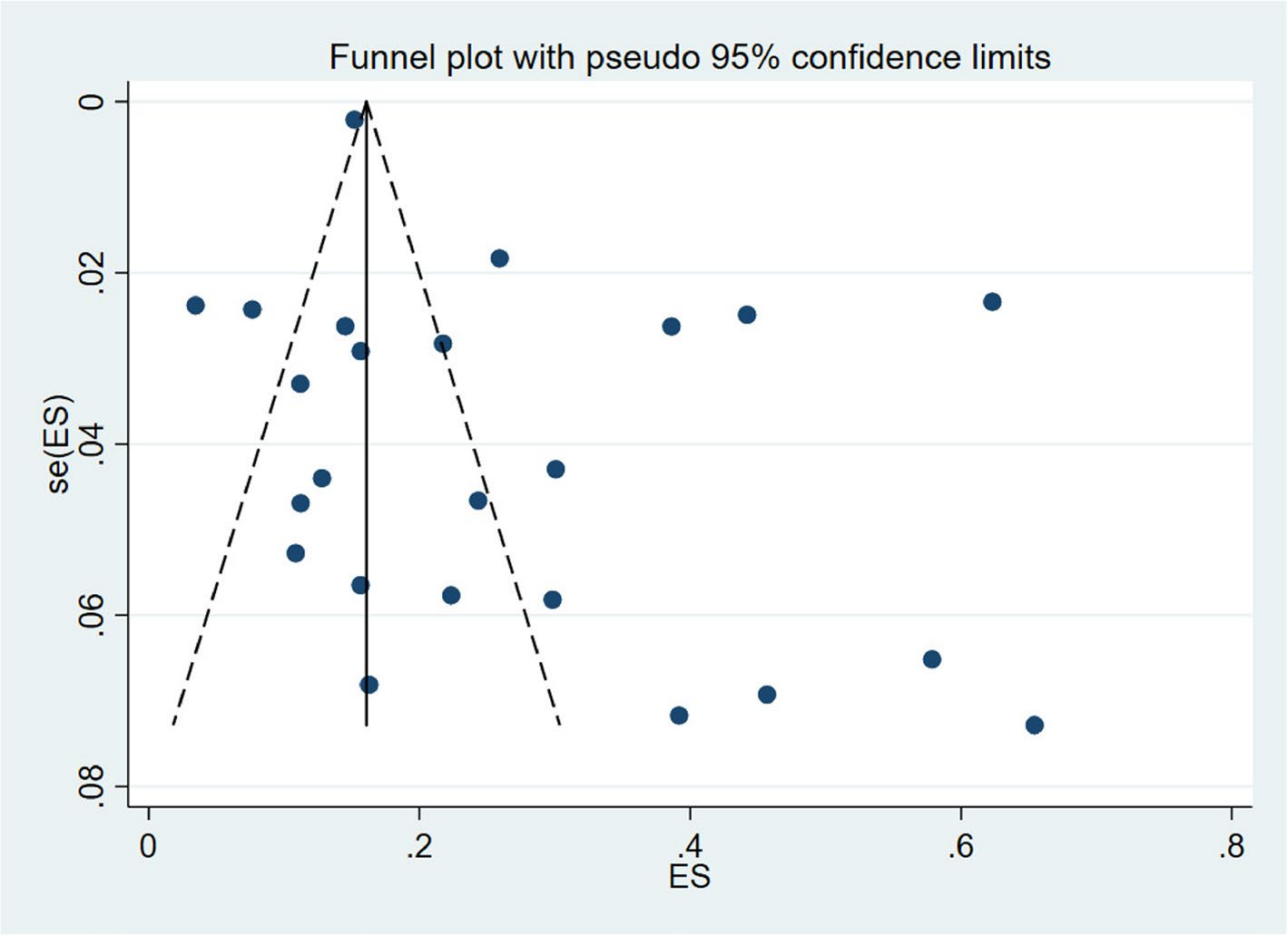
Funnel plot

**Fig. 4:**
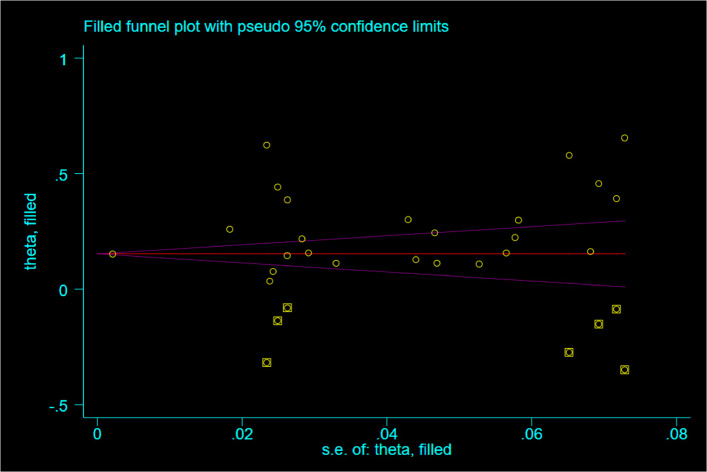
Trimming method

To assess the robustness of the results, a sensitivity analysis was conducted by sequentially excluding individual studies (Fig. 2). The estimated overall prevalence of social frailty fluctuated between 23.4% and 26.3%, showing no significant difference from the overall estimate, indicating that the results are relatively stable. A funnel plot (Fig. 3) and Egger’s test were used to assess publication bias. When all 24 studies were initially included, the funnel plot exhibited some asymmetry, and the Egger’s test results indicated significant publication bias (t = − 2.46, *p* = 0.022). To further assess the potential impact of this bias on the pooled effect size, we conducted a trim-and-fill analysis. The results showed that correcting the funnel plot asymmetry required adding 7 studies; the adjusted pooled effect size (increasing from 0.161 to 1.165) suggested that the original analysis may have overestimated the effect due to the absence of negative results. Given that the sample size of the study by Qi X et al. (*n* = 222,179) far exceeded that of other studies and might have significantly influenced the overall bias assessment, we conducted a sensitivity analysis after excluding this study. A redrawn funnel plot and Egger’s test indicated that publication bias was no longer significant (t = 0.18, *p* = 0.857). Applying the trim-and-fill method again, the results showed that no additional studies needed to be included; the pooled effect size was 0.257 (95% CI: 0.243–0.271) for the fixed-effects model and 0.270 (95% CI: 0.195–0.344) for the random-effects model, which were close to the pooled effect sizes before the initial exclusion. These results indicate that the meta-analysis findings are highly sensitive to the large-scale study by Qi X et al., and the publication bias detected in the original analysis primarily stemmed from this study with an exceptionally large sample size. After excluding its excessive influence, the body of evidence comprising the remaining 23 studies did not show significant publication bias, and the pooled effect sizes remained stable, suggesting that the core conclusions possess a certain degree of robustness (Fig. 4).

**Figure:**
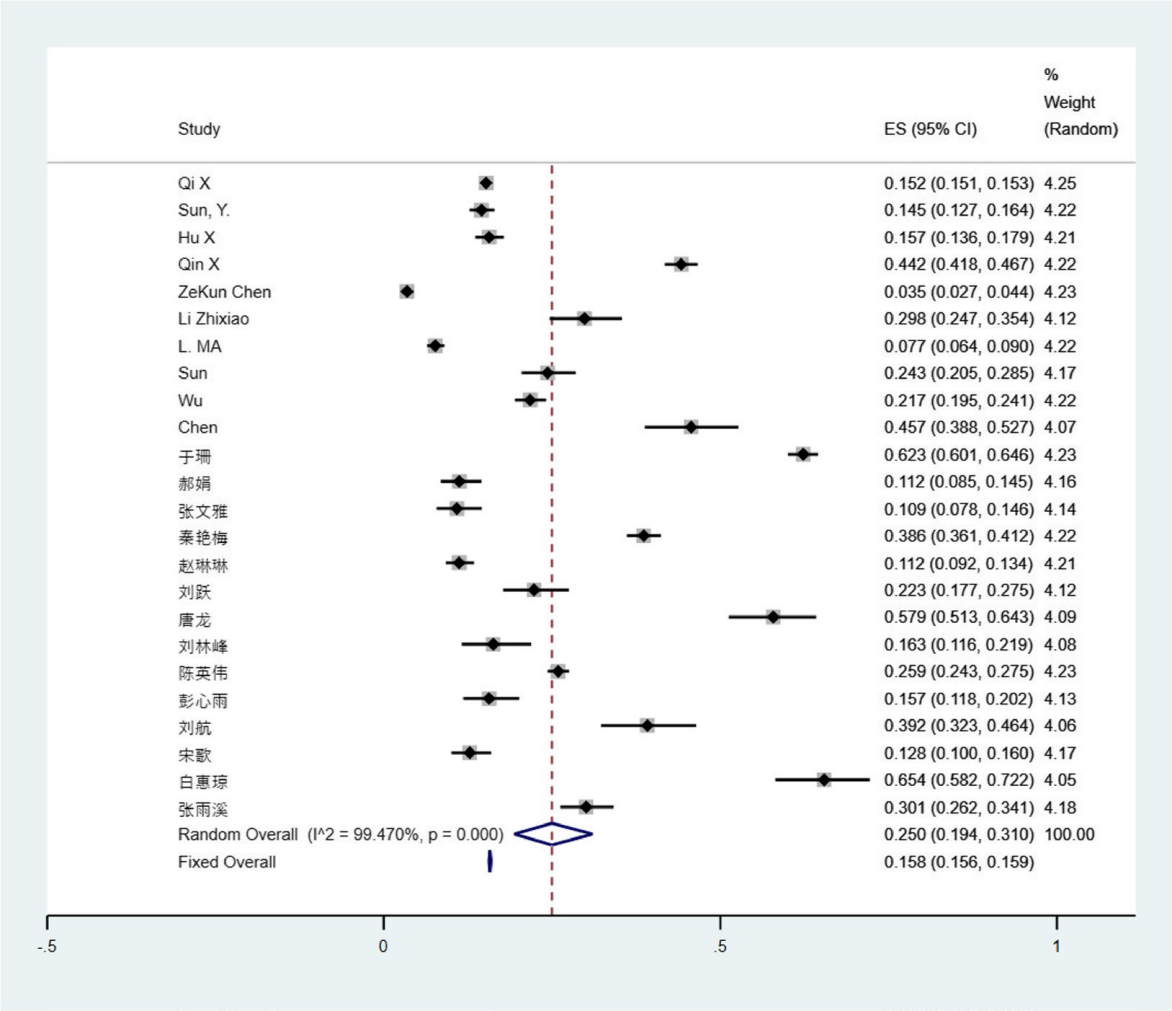
Forest plot of pooled prevalence

## Discussion

### Prevalence of social frailty in older adults

A meta-analysis of 24 original studies indicates that the pooled prevalence of social frailty among community-dwelling older adults in China is 25%. This suggests that social frailty is a significant health issue among the elderly. However, we found that the pooled estimates exhibited high heterogeneity (I^2^ = 99.47%, *P* < 0.001), indicating substantial differences across the included studies. This high heterogeneity directly affects the robustness and generalizability of our pooled estimate. Furthermore, our meta-analysis found that the combined prevalence of pre-social frailty among community-dwelling older adults in China was 42%, indicating that the population in the pre-stage is more extensive than those in a state of social frailty. This finding suggests that implementing screening and interventions for pre-social frailty among China’s community-dwelling older adults may provide a significant opportunity for effectively managing social frailty and improving health outcomes. However, we found that the pooled estimates exhibited high heterogeneity (I^2^ = 99.47%, *P* < 0.001), suggesting substantial differences in effect sizes among the included studies; therefore, the overall pooled estimate should be interpreted with caution. Therefore, we conducted a pre-specified subgroup analysis to elucidate the sources of heterogeneity and to more precisely characterize the distribution of social frailty across different subgroups. However, heterogeneity remained significant after subgroup analysis, and we ultimately chose a random-effects model to pool the prevalence estimates.

We first hypothesized that differences in diagnostic tools were a key methodological factor contributing to heterogeneity. Due to the lack of a standardized diagnostic tool for social frailty, the included studies utilized various instruments such as HALFT, MSFI, SFI, SFS, TFI, and SVI. Based on the differing theoretical frameworks underlying these diagnostic tools, we grouped diagnostic tools based on the theory of social need fulfillment—including HALFT, MSFI, SFI, and SFS—into one category, while classifying the remaining instruments as “other scales.”Among these, specialized social frailty scales based on the theory of social need satisfaction were used in 22 studies, with a combined prevalence of 24.2%, while studies using other scales had a combined prevalence of 52.4%. The effect size differed significantly between the different scale types (*p* = 0.001). In-depth analysis revealed that in the group of other scales, such as the TFI scale, social frailty is defined based on three sub-indicators: living alone, lack of social relationships, and lack of social support; however, there are currently no clear criteria for determining social frailty [30]. The SVI scale is a diagnostic tool developed based on the deficit accumulation model; diagnostic tools developed from different theoretical models may affect the accuracy of prevalence estimates. Currently, the HALFT scale is widely used in China for assessing social frailty, whereas other scales may not be specifically designed for this purpose or may lack a Chinese version [31]. This suggests that in future research and clinical screening, diagnostic tools for social frailty must be carefully selected and standardized to facilitate the comparison and integration of results. Furthermore, it is essential to develop or validate a comprehensive diagnostic tool suitable for the Chinese context that integrates both objective and subjective dimensions and encompasses social participation and social support.

Furthermore, our analysis revealed a significant difference between the pooled prevalence rates from cross-sectional studies (29%) and those from cohort studies (14%), with the difference between study types being statistically significant (*p* = 0.001). This indicates that study design influences prevalence results and may serve as an important source of heterogeneity. We attribute this phenomenon to the differing numbers of studies included in each group, which led to variations in prevalence estimates. Concurrently, our subgroup analysis based on study quality revealed no significant difference in pooled prevalence between the low-risk-of-bias and moderate-risk-of-bias groups (*P* = 0.277), suggesting that study quality is not a primary factor contributing to the high heterogeneity observed in this study.

However, it is important to note that while the subgroup analysis based on population characteristics showed no significant effect of gender on the effect size (*p* = 0.982), it revealed differences with significant public health implications. Subgroup analysis by gender showed that the combined prevalence of social frailty among older women was 22.7% (95% CI: 0.15–0.31), while that among older men was 22.6% (95% CI: 0.15–0.30). This finding is consistent with the conclusions of the study by Gong Xiaoyan et al. [32]. A possible reason is that women are more likely and more frequently express emotional experiences than men, leading to higher levels of loneliness among older women compared to older men, which in turn increases the risk of social frailty [33].

We divided the sample into urban and rural subgroups to conduct an in-depth, multi-level, and multi-faceted exploration of social frailty among community-dwelling older adults in China. The analysis showed that the pooled prevalence across 10 rural-based studies was 29.9% (95% CI: 0.20–0.30), while the pooled prevalence across 20 community-based studies was 25.6% (95% CI: 0.21–0.30). The difference in effect sizes between urban and rural areas (*p* = 0.440) was not significant, indicating that geographic location was not a major source of heterogeneity. The prevalence of social frailty among rural older adults was higher than that among urban community-dwelling older adults, a finding consistent with the conclusions of Yu Shan’s study [16]. The cause of this difference may lie in the fact that, as the urban–rural dichotomy has intensified and young and middle-aged rural residents have migrated en masse to cities for work, rural older adults—due to poor health status, low levels of family support, unstable economic resources, and a lack of fixed pensions—may have further widened the gap between rural and urban older adults in terms of social participation or social integration.

To investigate whether there are regional differences in the prevalence of social frailty, we conducted a subgroup analysis based on the study participants’ locations, dividing the study population into five subgroups: Central China, East China, North China, Southwest China, and other regions (primarily Northeast and Northwest China). The results indicate that there are differences in the pooled prevalence of social frailty across different regions of China. Among these, the “Other Regions” group had the highest overall prevalence at 32.1% (95% CI: 24.2%–40.0%), followed by North China at 24.4% (95% CI: 16.7%–32.0%) and East China at 23.1% (95% CI: 17.1%–29.0%). The pooled prevalence rates in Southwest and Central China were relatively lower, at 21.7% (95% CI: 17.3%–26.1%) and 19.5% (95% CI: 14.5%–24.5%), respectively. Our subgroup analysis generated the hypothesis that the prevalence of social frailty might vary by geographic region in China (e.g., potentially higher in the north). However, given the substantial unexplained heterogeneity and the limited number of studies in some regions, this finding is exploratory and should not be considered conclusive. Future research with standardized methodologies is needed to test this hypothesis. We believe that this regional disparity may be related to domestic labor migration trends, which are often driven by economic and educational factors. This migration has led to increasingly prominent aging issues in northern regions, directly weakening older adults’ social support networks and potentially exacerbating social frailty [34]. The limited number of studies included for certain regions (such as Southwest China and other areas), coupled with the lack of a nationally standardized social frailty assessment scale, may have affected the stability of the pooled results. Future research should focus on exploring the epidemiological characteristics and causes of social frailty among older adults in urban and rural areas and across different regions of China, with the aim of establishing unified assessment standards and comprehensively understanding its nationwide distribution.

Results from a stratified analysis based on publication year indicate that the prevalence of social frailty in China shows an increasing trend over time. The overall prevalence of social frailty was 15.5% between 2018 and 2022, rising to 32% between 2023 and 2025; the effect size differed significantly across publication year groups (*p* = 0.001), suggesting that temporal trends may be a source of heterogeneity. Only 8 studies were conducted between 2020 and 2022, involving a total of 8,284 participants; in contrast, 16 studies were conducted between 2023 and 2025, involving a total of 234,307 participants. The COVID-19 pandemic severely restricted older adults'’ social participation [35], with significant reductions in both physical and social activities, which may have further exacerbated the incidence of social frailty. More importantly, the substantial increase in sample size and a deeper understanding of the concept of social frailty may account for the higher prevalence estimates observed recently. We also note that among the 8 studies conducted between 2018 and 2022, 4 had sample sizes of fewer than 500; among the 16 studies conducted between 2023 and 2025, 7 had sample sizes of fewer than 500. We believe that the COVID-19 pandemic may have limited the survey scale of early studies to some extent, which may also partially explain why the combined prevalence rates from 2023 to 2025 were higher than those from the previous period.

Finally, even after conducting various subgroup analyses and meta-regressions, heterogeneity remained significant, suggesting the presence of unobserved confounding factors (such as differences in the definition of social frailty, sample representativeness, and levels of community services). This significant and not fully explained heterogeneity directly affects the robustness and generalizability of our pooled prevalence estimates. Given that the prevalence of social vulnerability reported across studies ranges from 3.5% to 65.4%, and considering differences in sample size, diagnostic tools, and study regions, the pooled estimate of 25.0% should be viewed as a statistical summary of highly diverse findings rather than an exact or universally applicable national rate. Therefore, this estimate is more suitable for generating hypotheses than for informing specific policy or clinical decisions. Therefore, this estimate is more suitable for generating hypotheses than for cautiously providing a basis for definitive policy or clinical decisions.

### Factors influencing social frailty in older adults

In summary, the factors influencing social frailty among older adults in the community primarily include demographic, health-related, and psychosocial factors. At the health level, physical frailty (OR = 4.15) and depression (OR = 2.82) showed the strongest associations. Physical frailty is significantly associated with social frailty, a finding consistent with previous studies [36]. Furthermore, research indicates that there is a bidirectional relationship between social frailty and physical frailty, and that this relationship changes with age [37]. A longitudinal study in Japan [38] noted that symptoms of physical frailty predict the future onset of social frailty; in particular, gait stability and muscle strength are considered important independent influencing factors for future social frailty. Maintaining and improving physical function through exercise, particularly balance and muscle strength, can serve as a key intervention strategy for addressing social frailty.

As a psychological factor, we found that depression is significantly associated with social frailty, consistent with previous research findings [39]. The negative emotions triggered by depression can affect older adults’ willingness and ability to participate in social activities, potentially reducing their social engagement and, consequently, the social support they receive.

Our study also indicates that demographic factors such as gender, marital status, and age are closely associated with social frailty. Older women face a 1.36-fold higher risk of social frailty compared to men, consistent with findings by Hyuma Makizako [40] and others. On the one hand, this may be attributed to physical symptoms (e.g., joint pain, insomnia, fatigue) and psychological symptoms (e.g., anxiety, depression) that emerge after menopause, which may limit older women’s capacity for social participation and lead to social frailty [41]. On the other hand, in terms of the division of household roles, older women typically shoulder more family caregiving responsibilities, which objectively consumes their time and energy that could be devoted to broader social interactions, leading to a gradual inward contraction and homogenization of their social support networks [17]. Future interventions could focus on the mental health and social participation of older women, encouraging them to maintain adequate time and space for socializing to reduce the risk of social frailty.

Furthermore, our study found that compared to community-dwelling older adults with spouses, those without spouses (including those who are divorced or widowed) face a 4.32-fold higher risk of social frailty. This aligns with the findings of Yang Xiaojiao et al. [42]. The underlying reason is that divorce or widowhood may lead to a reduction in social connections and a weakening of social support networks, thereby triggering or exacerbating feelings of loneliness. As an important emotional bond, the absence of a spouse may intensify older adults’ experience of loneliness and reduce the frequency and depth of their daily social interactions.

Although our study found that older adults aged ≥ 85 years had a 3.25-fold higher risk of social frailty compared to those aged 65–69 years, and this difference was statistically significant—a finding similar to that of Makizako et al. [43] —it cannot be denied that social frailty scores tend to increase with age. This may be due to the gradual decline in cognitive function with age, leading older adults to actively or passively reduce their participation in family activities and social interactions, thereby significantly increasing their risk of social isolation [44].

Regarding housing satisfaction as a psychosocial factor, our analysis also found that older adults dissatisfied with their living environment face a 2.13-fold increased risk of social frailty compared to those who are satisfied. We believe that since the primary sphere of activity for Chinese older adults after retirement is the home environment, the importance of their living conditions is particularly pronounced. Therefore, when selecting or planning housing for older adults, it is essential to prioritize age-friendly modifications to interior furnishings.

In summary, social frailty is the result of the combined effects of physical decline, psychological state, living environment, and macro-social structures, which aligns with Bunt’s core concept of the multidimensional nature of frailty. This also suggests that future intervention strategies must adopt a multi-level, integrated approach.

### Limitations

We also acknowledge the following limitations of this study: First, the meta-analysis of prevalence rates exhibited high heterogeneity. Although we conducted pre-specified subgroup analyses and meta-regression, and identified diagnostic tools, publication year, and study design as important moderating variables, some sources of heterogeneity remain unexplained. This heterogeneity affects the robustness and generalizability of the pooled estimates. Therefore, the pooled prevalence of 25.0% reported here should be regarded as the midpoint of a range of comprehensive estimates based on the available evidence, rather than an absolute precision. Second, meta-analyses of associated factors were limited by the data reported in the original studies, resulting in our ability to pool only a few factors. Furthermore, heterogeneity among studies regarding certain factors (such as marital status and physical frailty) remained high, suggesting that the relationship between these factors and the outcome may vary across different populations. Third, the absence of gray literature may lead to publication bias; although we verified the robustness of the results using the trim-and-fill method, we cannot completely rule out its impact on the overall effect estimate.

## Conclusion

This systematic review indicates that pre-social frailty and social frailty are notable concerns among community-dwelling older adults in China. Crucially, due to the very high heterogeneity across studies, the pooled prevalence estimates should be considered as a range of possible values rather than a precise figure, the exact prevalence requires further validation through high-quality research. The main influencing factors for social frailty include advanced age, female gender, being unmarried, depressive symptoms, dissatisfaction with housing, and coexisting physical frailty. Existing studies suggest that social support interventions and combined exercise and nutritional interventions may play a positive role in improving social frailty; future high-quality intervention studies should be conducted in Chinese populations to validate these findings [45]. In summary, public health and clinical practice should prioritize the systematic assessment and early management of social frailty among community-dwelling older adults. Efforts should focus on developing or validating social frailty diagnostic tools suitable for the Chinese elderly population, actively promoting relevant intervention studies and tracking long-term outcomes, and gradually establishing a scientific and effective intervention management system to enhance the overall quality of life for older adults.

## Supplementary Information

Supplementary Material 1.

## Data Availability

All data included in this analysis were extracted from publicly published studies. Detailed references for the incidence rates, effect sizes of risk factors, and their 95% confidence intervals from each original study are fully listed in the tables within the main text and in the appendices.

## References

[1] Qiao X. The past, present, and future of population aging in China. Social Policy Research. 2024;01:47–63+133.

[2] Gobbens RJ, van Assen MA, Luijkx KG, Wijnen-Sponselee MT, Schols JM. Determinants of frailty. J Am Med Dir Assoc. 2010;11(5):356–64. 20511103 10.1016/j.jamda.2009.11.008

[3] Bunt S, Steverink N, Olthof J, van der Schans CP, Hobbelen JSM. Social frailty in older adults: a scoping review. Eur J Ageing. 2017;14(3):323–34. 28936141 10.1007/s10433-017-0414-7 PMC5587459

[4] Page MJ, McKenzie JE, Bossuyt PM, Boutron I, Hoffmann TC, Mulrow CD, et al. The PRISMA 2020 statement: an updated guideline for reporting systematic reviews. Syst Rev. 2021;10(1):89. 33781348 10.1186/s13643-021-01626-4 PMC8008539

[5] Saltzman C. New FAI guidelines: STROBE, MOOSE, PRISMA, CONSORT. Foot Ankle Int. 2022;43(1):1. 35023395 10.1177/10711007211063029

[6] Qi X, Jia N, Hu J, Meng LB, Zeng P, Liu J, et al. Analysis of the status of social frailty in Chinese older adults with cardiovascular and cerebrovascular diseases: a national cross-sectional study. Front Public Health. 2023;11:1022208. 37293616 10.3389/fpubh.2023.1022208 PMC10244724

[7] Sun Y, Liu H, Li X, Zhang L, Xu W, Liu H, et al. Association of social frailty, sarcopenia, and oral frailty with depressive symptoms in Chinese older adults: a cross-sectional study. BMC Public Health. 2025;25(1):464. 39910522 10.1186/s12889-025-21662-2 PMC11796157

[8] Hu X, Liu H, Liu Q, Yuan T, Duan M, Luo Y, et al. Depressive symptoms and their influencing factors among older adults in China: a cross-sectional study. Front Public Health. 2024;12:1423391. 39618958 10.3389/fpubh.2024.1423391 PMC11605914

[9] Qin X, Liu H, Tao X, Zhou Z, Mei G, Zhang M, et al. Risk prediction model of frailty and its associated factors in older adults: a cross-sectional study in Anhui Province, China. Front Nutr. 2025;12:1611914. 40747332 10.3389/fnut.2025.1611914 PMC12310452

[10] Chen Z, Jiang X, Shi G, Wang Y, Chu X, Wang Z, et al. Psychogeriatrics. 2021;21(4):483–90. 33960060 10.1111/psyg.12696

[11] Li Z, Gu J, Li P, Hu J, Wang S, Wang P, et al. The relationship between social frailty and loneliness in community-dwelling older adults: a cross-sectional study. BMC Geriatr. 2024;24(1):73. 38238657 10.1186/s12877-024-04666-2 PMC10797967

[12] Ma L, Sun F, Tang Z. Social frailty is associated with physical functioning, cognition, and depression, and predicts mortality. J Nutr Health Aging. 2018;22(8):989–95. 30272104 10.1007/s12603-018-1054-0 PMC12876323

[13] Sun QQ, Tan K, Tang HY, Liu YY, Zhu H, Qin H, et al. Incidence and predictive value of social frailty among community-dwelling older adults in Southwest China: a prospective cohort study. Front Public Health. 2023;11:1103651. 36891342 10.3389/fpubh.2023.1103651 PMC9986618

[14] Wu KY, Chen DR, Chan CC, Yeh YP, Chen HH. Fear of falling as a mediator in the association between social frailty and health-related quality of life in community-dwelling older adults. BMC Geriatr. 2023;23(1):421. 37430231 10.1186/s12877-023-04144-1 PMC10334636

[15] Chen IC, Chang SH. The relationship between coronavirus-related anxiety and physical frailty, psychological frailty, and social frailty in community-dwelling older adults in Taiwan during the COVID-19 pandemic. 2023;37(4):260. 10.56808/2586-940X.1041.

[16] Yu Shan, Che Yajie, Su Biyinur Maimaiti, Wukele’ai Awutalipu, Guo Kaiyang, Miao Miao, Yan Ping. A Comparative Study on the Current Status of Social Frailty Among Urban and Rural Older Adults. J Nurs. 2025;40(15). 10.3870/j.issn.1001-4152.2025.15.006.

[17] Hao Juan, Fan Junyao, Wu Qiansheng. Survey on the Current Status of Social Frailty Among Community-Dwelling Older Adults in Hubei Province and Analysis of Influencing Factors. Contemporary Nurses (Late Edition). 2022;29(9).

[18] Zhang Wenya, Fan Junyao, Wang Tianying, Li Jie. Correlation Between Social Frailty and Depression Among Community-Dwelling Older Adults in Hubei Province. Practical Geriatrics. 2021;35(5).

[19] Qin Yanmei, Hao Yang, Shi Xuefei, Liu Yao, Zhao Yaning: Analysis of Influencing Factors and Pathways of Social Frailty Among Community-Dwelling Older Adults with Chronic Diseases Based on Social Ecological Theory. Nursing Research. 2025;39(18).

[20] Zhao L, Chang B, Hu Q, Du J, Shao S. Analysis of the correlation and influencing factors of physical, psychological, and social frailty in elderly patients with comorbidities. Zhonghua Quan Ke Yi Xue. 2025;24(6):670–8.

[21] Liu Yue. A Longitudinal Study onof Social Frailty and Short-Term Adverse Health Outcome among Older People in rural areas. Master’s Thesis. Shandong University of Traditional Chinese Medicine; 2024.

[22] Tang Long, Yang Jun, Zhang Yuanhui, Wang Yanni, Wei Shaojuan, Jin Haiqing, Mo Yadan. A Study on the Association Between Oral Frailty and Social Frailty in Community-Dwelling Older Adults with Type 2 Diabetes. General Practice Nursing. 2025;23(13).

[23] Liu Linfeng, Wang Hongyan, Cao Jun, Zhang Wen, Liang Qingfang, Ma Wenlian. A Study on the Correlation Between Self-Concealment, Perceived Benefits, and Social Frailty Among Community-Dwelling Older Adults with Type 2 Diabetes. Res Pract Health Care. 2024;21(7).

[24] Chen Yingwei, Huang Xinghui, Pu Yuhong, Xu Jixiang, Gao Junling. The Relationship Between Social Frailty and Quality of Life Among Community-Dwelling Older Adults. Geriatr Med Health Care. 2022;28(1).

[25] Peng Xinyu, Song Mingfang, Zhang Wan, Luo Bingbin, Chen Keyi, Wang Donghua. Current Status and Influencing Factors of Social Frailty Among Community-Dwelling Older Adults. Nursing Research. 2021;35(19).

[26] Liu H, Lin X. Current status and influencing factors of social frailty among community-dwelling older adults in Changsha. Occupational and Health. 2022;38(14):1978–81.

[27] Song Ge, Wang Ying, Gao Huanling, Chen Ling, Yang Yulin. The Association Between Sleep Disorders and Social Frailty. Pract Geriatr. 2022;36(8).

[28] Bai Huiqiong, Wei Pingping, Guo Jinrong, Liu Shunfang, Li Xiongbo, Huang Likun, Wu Hongxia, Sun Jianping. Current Status and Influencing Factors of Social Frailty Among Community-Dwelling Older Adults with Knee Osteoarthritis. In: Evidence-Based Nursing. vol. 9; 2023.

[29] Zhang Yuxi, Yu Weihua, Yang Xia, Deng Man: The Mediating Effect of Self-Rated Health on the Relationship Between Living Space and Social Frailty Among Community-Dwelling Older Adults. Chin J Pract Nursing. 2025;41(19).

[30] Iijima S, Ito A, Ito S, Sasaki T, Sugita Y. Trends in the assessment of social frailty in community-dwelling older adults: a scoping review. Cureus. 2024;16(8):e66614. 39258050 10.7759/cureus.66614 PMC11386154

[31] Zhang Lulu. Comparison of the Predictive Validity of Different Social Frailty Assessment Tools for Adverse Outcomes in Older Adults in Long-Term Care Facilities. Master’s Thesis. Chengdu University of Traditional Chinese Medicine; 2023.

[32] Gong X, Gao J, Hou C, You Q, He J, Bai D. Meta-analysis of the prevalence of social frailty in older adults. Military Nursing. 2023;40(04):98–102.

[33] Gu L, Wang M. Research progress on loneliness. Contemporary Nurses (Mid-Month Edition). 2024;31(06):26–30.

[34] Wu L, Huang Z, Pan Z. The spatiality and driving forces of population ageing in China. PLoS ONE. 2021;16(1):e0243559. 33428682 10.1371/journal.pone.0243559 PMC7799793

[35] Yamada M, Arai H. Understanding social frailty. Arch Gerontol Geriatr. 2023;115:105123. 37473693 10.1016/j.archger.2023.105123

[36] Liu Q, Huang Y, Gao S, Wang B, Li Y, Si H, et al. Joint trajectories of physical frailty and social frailty and associations with adverse outcomes: a prospective cohort study. Arch Gerontol Geriatr. 2024;122:105406. 38507855 10.1016/j.archger.2024.105406

[37] Wu F, Hu Y. Research progress on the relationship between social frailty and other frailty subtypes. Chinese Journal of Clinical Health Care. 2025;28(03):424–8.

[38] Nagai K, Tamaki K, Kusunoki H, Wada Y, Tsuji S, Itoh M, Sano K, Amano M, Hayashitani S, Yokoyama R, et al. Physical frailty predicts the development of social frailty: a prospective cohort study. 2020;20(1):403. 10.1186/s12877-020-01814-2 PMC7557012 33054731

[39] Qi X, Li J. The Relationship between Social Frailty and Depressive Symptoms in the Elderly: A Scoping Review. Int J Environ Res Public Health. 2022;19(24). 10.3390/ijerph192416683 PMC9779347 36554564

[40] Makizako H, Shimada H, Doi T, Tsutsumimoto K, Hotta R, Nakakubo S, Makino K, Lee S. Social Frailty Leads to the Development of Physical Frailty among Physically Non-Frail Adults: A Four-Year Follow-Up Longitudinal Cohort Study. Int J Environ Res Public Health. 2018;15(3). 10.3390/ijerph15030490 PMC5877035 29534470

[41] Le Y. Survey and correlation analysis of personality traits, psychological resilience, and the incidence of postmenopausal symptoms in older women. Chin J Mater Child Health. 2021;36(10):2337–9.

[42] Yang X, Wang F, Zhang X, Xing F. The impact of family care and social support on health-promoting behaviors among older adults. Chin J Public Health. 2018;34(09):1266–9.

[43] Makizako H, Tsutsumimoto K, Shimada H, Arai H. Social frailty among community-dwelling older adults: recommended assessments and implications. Ann Geriatr Med Res. 2018;22(1):3–8. 32743237 10.4235/agmr.2018.22.1.3 PMC7387641

[44] Wang X, Han Y, Fan R, Zhao Y, Liu Y, Li Y, et al. The impact of family care and emotional balance on social isolation among community-dwelling older adults. Nurs Res. 2020;34(07):1266–8.

[45] Wu Y, Sun Q, Cui L. Current status of social frailty among older adults and advances in intervention research. Chinese Journal of Geriatric Health Care. 2024;22(04):126–9.

